# Transition of Radioactive Cesium Deposition in Reproductive Organs of Free-Roaming Cats in Namie Town, Fukushima

**DOI:** 10.3390/ijerph18041772

**Published:** 2021-02-11

**Authors:** Yohei Fujishima, Yasushi Kino, Takumi Ono, Valerie Swee Ting Goh, Akifumi Nakata, Kentaro Ariyoshi, Kosuke Kasai, Tadashi Toyoda, Toru Akama, Hirofumi Tazoe, Masatoshi Yamada, Mitsuaki A. Yoshida, Tomisato Miura

**Affiliations:** 1 Department of Radiation Biology, Tohoku University School of Medicine, 2-1 Seiryo-machi, Aoba-ku, Sendai, Miyagi 980-8575, Japan; yohei.fujishima@med.tohoku.ac.jp; 2 Department of Chemistry, Tohoku University Graduate School of Science, 6-3 Aramaki Aza-Aoba, Aoba-ku, Sendai, Miyagi 980-8578, Japan; yasushi.kino.e5@tohoku.ac.jp (Y.K.); takumi.ono.s4@dc.tohoku.ac.jp (T.O.); 3 Department of Bioscience and Laboratory Medicine, Hirosaki University Graduate School of Health Sciences, 66-1 Hon-cho, Hirosaki, Aomori 036-8564, Japan; h19gg801@hirosaki-u.ac.jp (V.S.T.G.); kokasai@hirosaki-u.ac.jp (K.K.); 4 Department of Pharmacy, Faculty of Pharmaceutical Science, Hokkaido University of Science, Sapporo, 7-Jo 15-4-1 Maeda, Teine, Sapporo, Hokkaido 006-8590, Japan; nakata_a@hus.ac.jp; 5 Integrated Center for Science and Humanities, Fukushima Medical University, 1 Hikariga-oka, Fukushima 960-1295, Japan; ariyoshi@fmu.ac.jp; 6 Toyoda Animal Hospital, 402-7 Kanairo, Nihonmatsu, Fukushima 964-0915, Japan; tadashit@violin.ocn.ne.jp; 7 Akama Industry Co., Ltd., 259-3 Babauchi, Kakura, Namie, Fukushima 979-1536, Japan; kra9791536@yahoo.co.jp; 8 Department of International Cooperation and Collaborative Research, Institute of Radiation Emergency Medicine, Hirosaki University, 66-1 Hon-cho, Hirosaki 036-8564, Japan; tazoe@hirosaki-u.ac.jp; 9 Central Laboratory, Marine Ecology Research Institute, 300 Iwawada, Onjuku, Isumi, Chiba 299-5105, Japan; m-yamada@kaiseiken.or.jp; 10 Institute of Chromosome Life Science, 11-5-409, Fukuokachuo 2-Chome, Fujimino-shi, Saitama 356-0031, Japan; mtak_yoshidad1955@axel.ocn.ne.jp; 11 Department of Risk Analysis and Biodosimetry, Institute of Radiation Emergency Medicine, Hirosaki University, 66-1 Hon-cho, Hirosaki, Aomori 036-8564, Japan

**Keywords:** Fukushima, free-roaming cat, radioactive cesium, reproductive organ, internal contamination

## Abstract

We investigated the internal contamination by radioactive cesium associated with the FDNPP accident, in the testes or uterus and ovaries of free-roaming cats (*Felis silvestris catus*), which were protected by volunteers in the Namie Town, Fukushima. A total of 253 samples (145 testes and 108 uterus and ovaries) obtained from adult cats and 15 fetuses from 3 pregnant female cats were measured. Free-roaming cats in Namie Town had a higher level of radioactive contamination in comparison to the control group in Tokyo, as the ^134^Cs + ^137^Cs activity concentration ranged from not detectable to 37,882 Bq kg^−1^ in adult cats. Furthermore, the radioactivity in the fetuses was almost comparable to those in their mother’s uterus and ovaries. The radioactivity was also different between several cats protected in the same location, and there was no significant correlation with ambient dose-rates and activity concentrations in soil. Moreover, radioactive cesium levels in cats decreased with each year. Therefore, it is likely that decontamination work in Namie Town and its surroundings could affect radioactive cesium accumulation, and thus possibly reduce the internal radiation exposure of wildlife living in contaminated areas. It is hence necessary to continue radioactivity monitoring efforts for the residents living in Namie Town.

## 1. Introduction

After the accident in the TEPCO Fukushima Daiichi Nuclear Power Plant (FDNPP), the surrounding areas were contaminated by large amounts of released radionuclides [1,2]. A 20-km radius around the FDNPP was initially established as a restricted zone to avoid unnecessary radiation exposure to residents. As the evacuation order was issued with no advanced warning, all residents living within the 20-km radius had to evacuate immediately without packing and were not allowed to bring along their companion animals during evacuation. Emergency animal shelters were launched in Iino Town, Fukushima Prefecture in April 2011 and in Miharu Town, Fukushima Prefecture in October 2011, to rescue companion animals left behind at the time of evacuation. Large-scale trap and rescue operations for cats were also performed several times by the Ministry of Environment, local government, and local veterinary medical association. Despite multiple efforts to rescue abandoned companion animals, cats (*Felis silvestris catus*) in particular have been reproducing in the restricted zones and Namie Town [3]. Since September 2013, volunteers in Namie Town are managing abandoned (now free-roaming) cats from uncontrolled reproduction and possible disruption to the area’s ecosystem, using the “Trap-Neuter-Vaccinate-Return (TNVR) [4]” program.

With regards to radiation, although the ambient dose-rates are gradually decreasing, prolonged effects of chronic low-dose exposure on animals are expected to be seen in the coming years. Therefore, by assessing biological effects from radioactive substances, we are able to understand any possible health effects caused by radiation. To date, several species of animals were evaluated (livestock bulls [5], wild boars [6,7,8], wild Japanese monkeys [9,10,11], wild rodents [12,13,14] and freshwater fish [15,16,17]) around the evacuation zone of the FDNPP. Furthermore, some studies [18] suggested that some negative biological effects seen in wild animals were likely caused by the release of radionuclides from the FDNPP accident.

Decontamination efforts gradually started from October 2013 to remove radioactive substances released by the FDNPP accident, such that the impact of environmental pollution and radiation exposure on human health and the surrounding environment could be reduced. For example, decontamination work in urban areas involves the removal of contaminated topsoil (0–5 cm) and replacing the surface with non-contaminated soil. As a result, evacuation orders in some parts of Namie Town were able to be lifted on 31 March 2017, due to low ambient dose-rates after decontamination. However, there are fewer reports focusing on the biological effects before and after decontamination work. Moreover, in existing research, the deposition of the radioactive substances in wild animals was not studied in urban areas. Although radioactive substances were heterogeneously distributed, detailed surveys of privately owned land would be difficult in urban areas when evacuated residents return in the future.

Radioactive substances released by the FDNPP accident includes radioisotopes of iodine (^131^I, ^132^I and ^133^I), cesium (^134^Cs, ^136^Cs and ^137^Cs), tellurium (^132^Te), and inert gases (such as ^133^Xe) [19,20]. These radionuclides can contribute to ambient dose-rates and be potential health risks immediately after the accident. Among these radionuclides, radioactive cesium has longer half-lives (^134^Cs, *t*_1/2_ = 2.06 years; ^137^Cs, *t*_1/2_ = 30.1 years), and are most likely the major contributors to radioactive contamination from 2013 to 2016. Hence, in this study, we monitored radioactive cesium (^134^Cs and ^137^Cs) derived from the FDNPP accident, in free-roaming cats caught in urban areas. We also measured the ambient dose-rates and soil cesium levels to verify the effectiveness of decontamination work and to evaluate how decontamination work changes the deposition of radioactive cesium in animals. In addition, we also evaluated feline leukemia virus (FeLV) and feline immunodeficiency virus (FIV) infections in free-roaming cats, as they are among the most common infectious diseases in domestic cats and are known to cause immunosuppression.

## 2. Materials and Methods

### 2.1. Animal Collection

**Figure 1:**
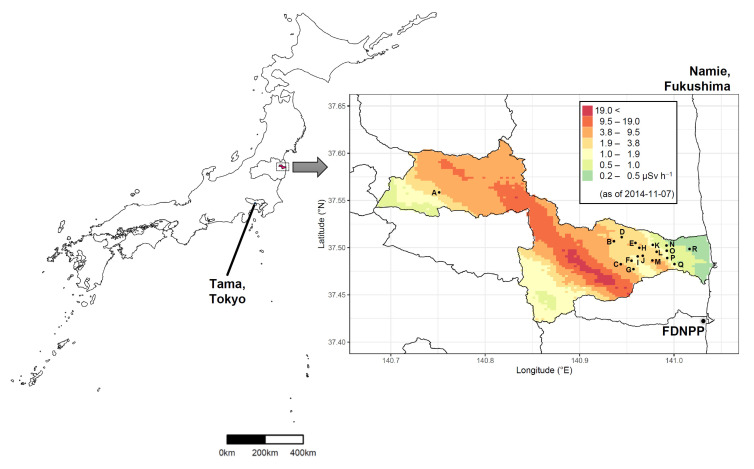
Sampling areas of free-roaming cats protected in Namie Town, Fukushima, and Tama area, Tokyo. The magnified map shows Namie Town and the sampling areas [(A) Tsushima, (B) Murohara, (C) Suenomori, (D) Tatsuno, (E) Karino, (F) Tajiri, (G) Obori, (H) Kakura, (I) Midorigaoka, (J) Uenohara, (K) Sakata, (L) Kawazoe, (M) Ushiwata, (N) Nishidai, (O) Gongendo, (P) Hiwatashi, (Q) Takase and (R) Kiyohashi], as well as the TEPCO Fukushima Daiichi Nuclear Power Plant (FDNPP). The maps were modified shapefiles from the National Land Numerical Information download service (data was retrieved from http://nlftp.mlit.go.jp/ksj/index.html on 5 February 2016). The heat map shows the ambient dose-rates on 7 November 2014 provided by the Nuclear Regulation Authority 9th airborne monitoring survey (data were retrieved from http://radioactivity.nsr.go.jp/ja/list/362/list-1.html on 5 February 2016).

Free-roaming cats in Namie Town were caught in humane live traps from September 2013 for TNVR [4]. Through surgical castration or ovariohysterectomy, reproductive organs, such as testes or uterus and ovaries, were extracted. In this study, we analyzed 253 samples (145 testes and 108 uterus and ovaries) from free-roaming cats rescued in October 2013 to December 2016 from 18 areas in Namie Town (Figure 1, Table 1). As a control, 10 samples (5 testes and 5 uterus and ovaries) from spayed and neutered free-roaming cats in Tama-area, Tokyo were also analyzed (Figure 1, Table 1). Samples were stored at –20 °C, until radioactivity measurements were performed.

### 2.2. Tests for FeLV and FIV

To evaluate FeLV and FIV, blood from the cephalic antebrachial vein was used. Viral infections were detected with commercial immunochromatography kits of Checkman FeLV (Kyoritsu Seiyaku Corporation/Adtec, Tokyo, Japan) and Checkman FIV (Kyoritsu Seiyaku Corporation/Adtec, Tokyo, Japan).

### 2.3. Ambient Dose-Rate Measurements

Ambient dose-rates were measured at 4–5 points in a 20 m^2^ area centered around the location, where the feline trap was installed on 30–31 July 2014, 22–23 July 2015, and 10–11 September 2016, using a NaI(Tl) scintillation survey meter (TCS-171B, Hitachi Aloka Medical, Ltd., Tokyo, Japan). The measurements were expressed as micro-grays per hour at 1 m above the ground with the time constant of the survey meter set to 10 s. Measurements were recorded after a minimum wait of 30 s for the readings to be stabilized. The ambient dose-rate in Tama area was referenced from the nearest monitoring post data provided by Nuclear Regulation Authority, Japan (data was retrieved from https://radioactivity.nsr.go.jp/map/ja/download.html on 15 March 2017).

### 2.4. Measurements of Activity Concentrations in Reproductive Organ and Soil Samples

For reproductive organs, samples were homogenized in separate plastic containers (U-8 container, SANPLATEC Co., Ltd., Osaka, Japan) after thawing. As for the soil samples, five surface soil samples of 5 cm depth were collected at each sampling point. The soil samples were completely dried at 120 °C, for 20 h before measurement. Each sample was transferred into separate U-8 plastic containers. The weight and height of the samples in the U-8 container was measured in order to calculate the sample density.

### 2.5. Gamma-Spectrometry

The activity concentration for the collected samples was determined by gamma-ray spectrometry, using a hyperpure germanium (HPGe) detector (ORTEC GEM-40190, SEIKO-EG&G Co., Ltd., Tokyo, Japan), as previously shown by Fukuda et al. [21]. ^134^Cs and ^137^Cs were detected using 604.6 and 795.8 keV gamma-ray energies, respectively, to satisfy measurement uncertainty from counting statistics to below 5% of the corresponding activity concentration. Activity was decay-corrected to the sampling date, and activity concentration was calculated as per kilogram of dry weight of the soil samples.

### 2.6. Statistics

Correlation analysis was carried out by calculating the Pearson’s product-moment correlation coefficient or Spearman’s rank correlation coefficient, based on Shapiro-Wilk normality tests. Wilcoxon rank sum test was applied to assess differences between two groups. The results were considered statistically significant if *p*-values below 0.05 were obtained. All statistical analyses were performed using the R version 4.0.3 (R Development Core Team, Austria) [22].

## 3. Results

### 3.1. Ambient Dose-Rates and Radioactive Cesium Activity Concentrations in Soil at Namie Town

**Figure 2:**
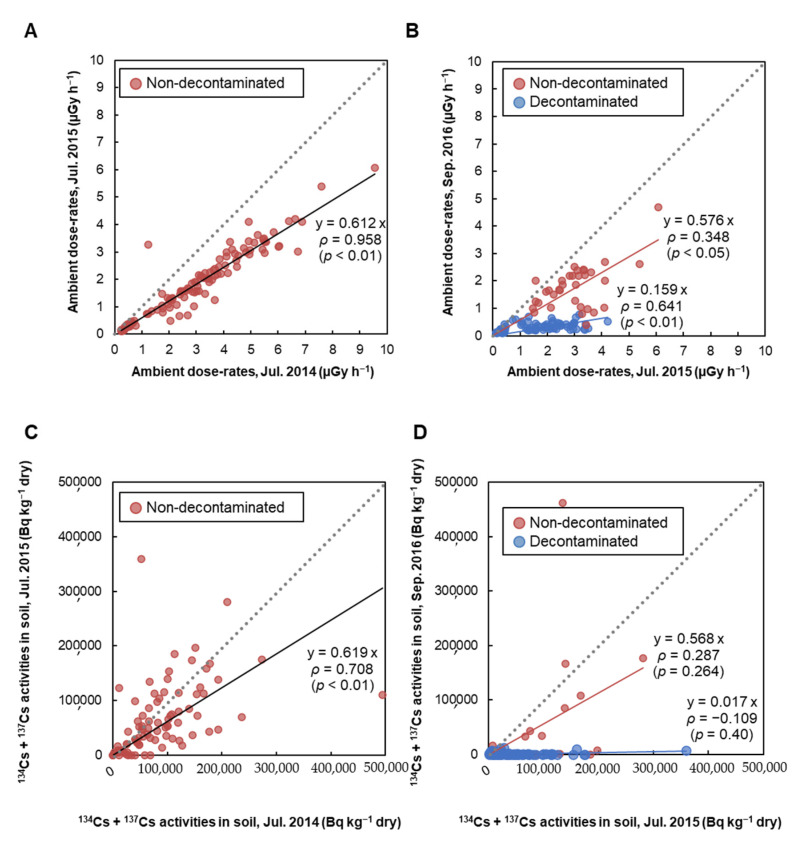
Ambient dose-rates and ^134^Cs + ^137^Cs activity concentrations in soil. Comparison of ambient dose-rates measured in (**A**) 2014 vs. 2015, (**B**) 2015 vs. 2016, and of ^134^Cs + ^137^Cs activity concentrations in soil measured in (**C**) 2014 vs. 2015, (**D**) 2015 vs. 2016. Red and blue circles represent areas that are non-decontaminated and decontaminated, respectively. Decontamination work in our research location in Namie Town started from September 2015.

Ambient dose-rates and radioactive cesium (^134^Cs + ^137^Cs) activity concentrations in soil of specimen collection sites were represented in Figure 2A (2014 vs. 2015), Figure 2B (2015 vs. 2016), Figure 2C (2014 vs. 2015), Figure 2D (2015 vs. 2016), respectively. In particular, decontamination work was performed only in 2015 and 2016. The ambient dose-rates in Namie Town in July 2015 decreased by approximately 39%, as compared to July 2014 (Figure 2A). Comparing July 2015 and September 2016, the ambient dose-rates decreased sharply in the decontaminated areas, with an average decrease of 62%. On the other hand, even in the areas where decontamination was not performed, the ambient dose-rates decreased moderately to an average of 38% (Figure 2B). A similar tendency was observed for ^134^Cs + ^137^Cs activity concentration in soil, but its distribution was extremely heterogeneous. ^134^Cs + ^137^Cs activity concentration in soil at July 2015 decreased by approximately 40%, as compared to July 2014 (Figure 2C). In the decontaminated areas, ^134^Cs + ^137^Cs activity concentration in soil significantly reduced by 90% in September 2016 as compared to July 2015. However, in areas where decontamination was not carried out, the average decrease was 44%, and if the points where low ^134^Cs + ^137^Cs activity concentration was omitted, the average rate of decrease was only 5% (Figure 2D). In addition, a strong correlation was observed between ambient dose-rate and ^134^Cs + ^137^Cs activity concentration in soil (2014: *r* = 0.67, *p* < 0.01; 2015: *r* = 0.68, *p* < 0.01; 2016: *r* = 0.60, *p* < 0.01).

### 3.2. Infection of FIV/FeLV

Immunochromatography tests for FIV and FeLV were performed on 211 cats. Fifteen cats (7.1%) were positive for FIV antigen and 16 cats (7.6%) were positive for FeLV antibody. Only 2 cats were positive for both FIV and FeLV antigens.

### 3.3. Radioactive Cesium Activity Concentration in Testes/Uterus and Ovaries

**Figure 3:**
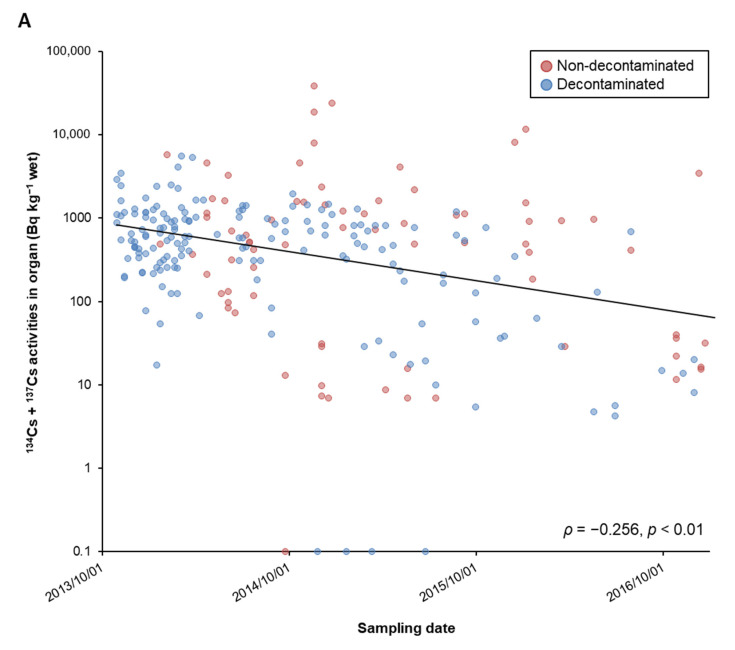

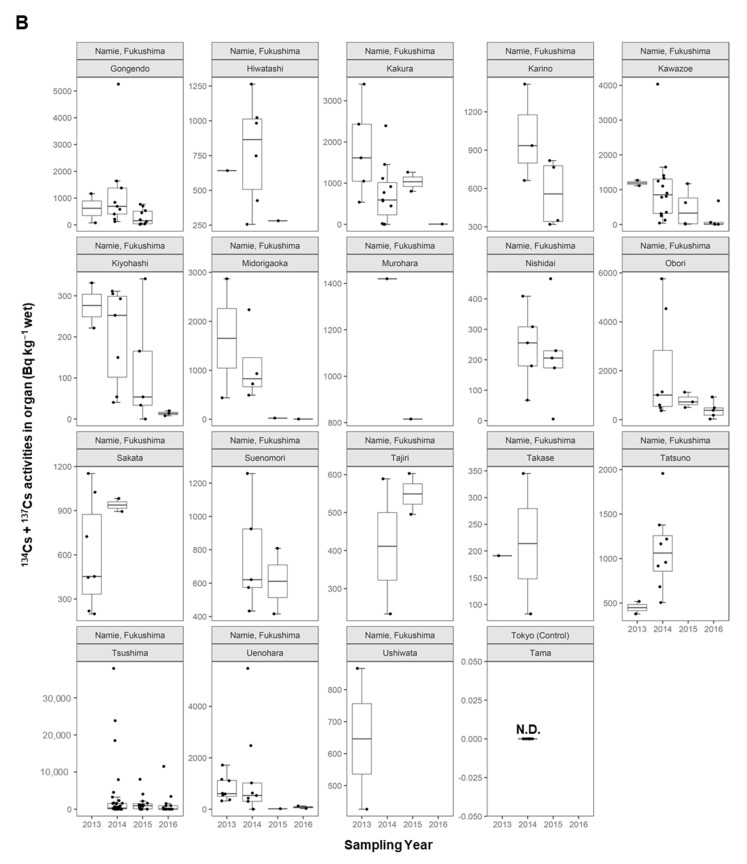
Change in ^134^Cs + ^137^Cs activity concentrations in reproductive organs of cats, with respect to (**A**) date caught and (**B**) area caught. In panel (**B**), each boxplot represents its distribution and black dots shows individual ^134^Cs + ^137^Cs activity concentrations in reproductive organs. N.D.; not detected.

There was no macroscopic abnormality in the testes/uterus and ovaries. Gamma spectrometry was performed in uterus and ovaries (108 samples) and testes (145 samples) of free-roaming cats captured in both non-decontaminated and decontaminated areas in Namie Town. ^134^Cs + ^137^Cs activity concentration accumulated in each organ ranged from not detectable to the maximum of 37,882 Bq kg^−1^ (Figure 3A). Cats from Hiwatashi, Kiyohashi, Nishidai, Sakata, and Ushiwata areas showed low ^134^Cs + ^137^Cs activity concentrations of 1500 Bq kg^−1^ or less, while cats with high ^134^Cs + ^137^Cs activity concentrations were seen in Gongendo, Uehara, Kawazoe, Midorigaoka, and especially, in Tsushima areas (Figure 3B). Moreover, ^134^Cs + ^137^Cs activity concentrations differed greatly even in cats caught in the same collection area. As ^134^Cs + ^137^Cs activity concentrations were monitored in cats from October 2013 to December 2016, the half-life was approximated to 310 days (Figure 3). On the other hand, all 10 cats from Tama area of Tokyo, which served as the control area, showed radioactive cesium activity concentrations below the detection limit (16.6 Bq kg^−1^, maximum 35.7 Bq kg^−1^, minimum 10.6 Bq kg^−1^), and were significantly lower than cats in Namie Town, Fukushima (*p* < 0.01).

**Figure 4:**
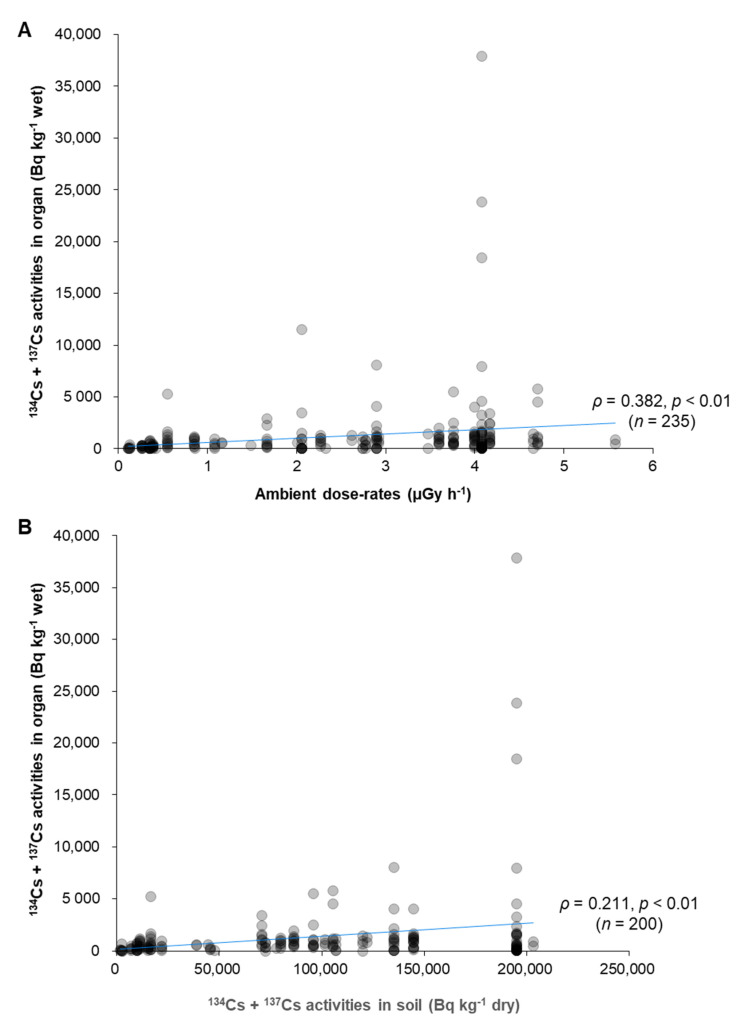
^134^Cs + ^137^Cs activity concentrations in reproductive organs of cats compared with (**A**) ambient dose-rates and (**B**) ^134^Cs + ^137^Cs activity concentrations in soil. Grey dots represent individual ^134^Cs + ^137^Cs activity concentrations in reproductive organs.

There was also no clear correlation between the areas of where the cat was captured (ambient dose-rates and ^134^Cs + ^137^Cs activity concentration in soil) and ^134^Cs + ^137^Cs activity concentration in the reproductive organs of cats (Figure 4).

**Figure 5:**
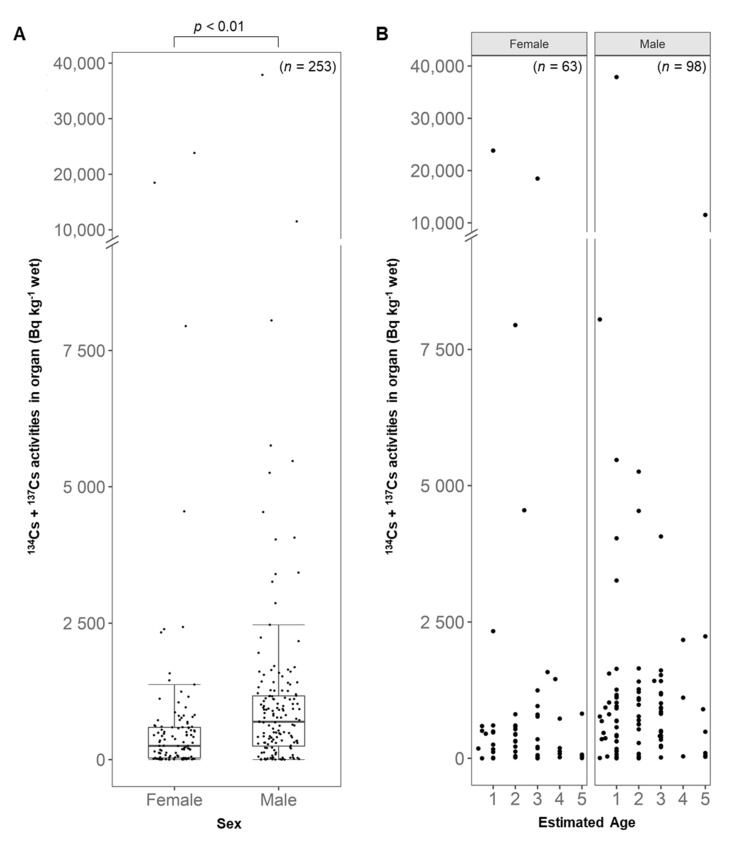
(**A**) An overall comparison and (**B**) a comparison with available estimated age of ^134^Cs + ^137^Cs activity concentrations in reproductive organs between male and female cats. Boxplot represents its distribution and black dots shows individual ^134^Cs + ^137^Cs activity concentrations in reproductive organs.

When ^134^Cs + ^137^Cs activity concentration in testes and uterus and ovaries were compared, ^134^Cs + ^137^Cs activity concentration was higher in male than female cats (*p* < 0.01, Figure 5A). In addition, some age-dependency was observed as younger male cats tended to have a higher accumulation of radioactive cesium in their testes (Figure 5B).

### 3.4. Radioactive Cesium Activity Concentrations Compared between Mother and Fetuses

**Figure 6:**
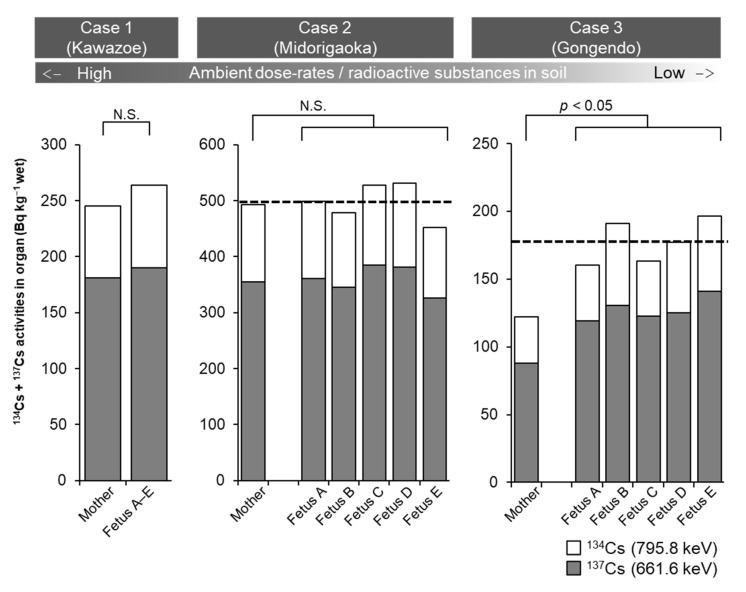
Comparison of ^134^Cs + ^137^Cs activity concentrations in the cat maternal uterus and fetuses. Dashed line in Case 2 and 3 represents mean activity concentrations of fetuses.

In this study, we were also able to compare ^134^Cs + ^137^Cs activity concentrations in mother’s uterus and fetus, as three female cats (caught in February 2014) were discovered to be pregnant while spaying. Only Case 3 (caught in Gongendo area) showed a significantly higher ^134^Cs + ^137^Cs activity concentration in the fetus than the maternal uterus. In the other two cases (caught in Kawazoe and Midorigaoka areas), there was no difference in ^134^Cs + ^137^Cs activity concentration between the fetus and maternal uterus (Figure 6). There was also no association seen between the areas of where the cats were caught (ambient dose-rate and ^134^Cs + ^137^Cs activity concentrations in the soil) and accumulated ^134^Cs + ^137^Cs activity concentrations in the fetus and maternal uterus.

## 4. Discussion

Due to the sudden evacuation orders issued to Namie Town residents after the FDNPP accident, the evacuees had to leave their companion animals behind. Companion animals including cats left behind at the time of the evacuation were rescued and neutered by various organizations [3]. However, many free-roaming cats are still found in Namie Town and its surroundings. As evacuation orders are slowly lifting and more residents are returning, population control of free-roaming cats is thus necessary. As proper management of cat population control is required, a volunteer group in Namie Town is now continuing the initial efforts previously carried out by the local government, as recommended by the Ministry of the Environment’s Guidelines [23].

No notable deformities such as malformations or growth retardations in reproductive organs of cats were seen with macroscopic observations. In addition, FeLV and FIV infections, which are common infectious diseases in domestic cats, were 7.1% and 7.6% respectively. In comparison to previous reports (FeLV: 0–8.1%; FIV: 0–12.8% [24,25,26,27,28,29]), FeLV and FIV infection rates in Namie Town were very similar to other non-contaminated areas. In Minamisoma City, dogs were reported to be aggressive and to bite humans. Their aggressiveness could be attributed to intermittent aftershocks and mental stress caused by owner abandonment as a result of immediate evacuation [30]. In contrast, cats rescued in Namie Town were not wary of us based on personal observations. Moreover, as the infection rates of FeLV and FIV were comparable to other general populations of domestic cats, the health of free-roaming cats was well controlled in Namie Town.

From our results, we showed that radioactive cesium activity concentrations in reproductive organs of cats was higher than that of the control area. With regards to the dynamics of radioactive cesium in the body, a model was proposed where radioactive cesium absorbed in the body through the digestive tract was transferred to visceral tissues via blood and finally excreted as urine [31]. Even though reproductive organs were only analyzed in this study, we could also expect radioactive cesium accumulation in other unanalyzed organs. According to a report in radioactive cesium distribution in cattle after the FDPP accident [5], activity concentrations were higher in skeletal muscle, kidney, and liver, as compared to blood, despite individual variations. In addition, as the testis showed the same activity concentration as the kidney and liver [5], it was thus highly likely that high radioactive cesium would be present in the skeletal muscle of free-roaming cats. Furthermore, the activity concentration of the feline testes was higher than that of the uterus and ovaries. Some studies also showed the same tendency in pigs and wild boars [32,33], but the underlying reason why ^134^Cs + ^137^Cs activity concentrations between males and females differed so markedly remains unclear. Furthermore, age effect was not observed on the cesium concentration in cats, although some young cats showed high radioactive cesium concentrations in both testes and uterus and ovaries. Wada et al. reported that the biological half-life of the ^137^Cs in cats was 30.8 days [34]. In this study, the youngest cat analyzed was 3-months old (3 biological half-lives elapsed). Therefore, we speculated that a high amount of radioactive cesium in young cats was likely not dependent on mother–fetus transition.

A slight correlation was seen when ambient dose-rates was compared with ^134^Cs + ^137^Cs activity concentrations in reproductive organs, but no correlation was seen with ^134^Cs + ^137^Cs activity concentrations in the soil. The weak correlation could be due to (1) heterogeneous contamination by radioactive substances in different habitats, (2) wide home range and movement between habitats (including movement between decontaminated and non-decontaminated areas), and (3) uncontaminated cat food fed to rescued cats. According to previous reports, the home range of cats could range from several hundred meters to several kilometers [35,36,37,38], suggesting that extreme outliers of highly contaminated cats caught in some areas (e.g., Uenohara and Gogendo areas in 2014) could be due to cat movement. Furthermore, as decontamination work was carried out in each administrative district, radioactive cesium accumulation in the body could also be affected when a cat moves between non-decontaminated and decontaminated areas.

Moreover, the transfer of radionuclides from mother to fetus was one of the major concerns of exposure to internal radiation. As radioactive cesium was also detected in the fetus, this finding suggests that cesium was able to transfer freely from mother to fetus.

## 5. Conclusions

Since October 2013, we have been continuously monitoring radioactive cesium activity concentrations in reproductive organs of free-roaming cats in Namie Town. We showed that radioactive cesium levels decreased with each year. Therefore, it is likely that decontamination work in Namie Town and its surroundings could affect radioactive cesium accumulation, and thus possibly reduce the internal radiation exposure of wildlife living in contaminated areas. These findings suggest that analyzing radioactive substance deposition in animals and soil is useful to evaluate the effectiveness of decontamination work and to monitor the environment of urban areas.

## Figures and Tables

**Table 1:** Locations and decontamination status of sampling areas.

Sampling Areas		GPS Location		Distance from	Decontamination
		Longitude (°)	Latitude (°)	F1-NPP (km)	Status ^†^
Namie, Fukushima	(A) Tsushima	37°33′30.8″ N	140°45′04.4″ E	29.1	Non-decontaminated
	(B) Murohara	37°30′24.4″ N	140°56′11.0″ E	12.6	Decontaminated
	(C) Suenomori	37°28′56.9″ N	140°56′37.0″ E	10.3	Non-decontaminated
	(D) Tatsuno	37°30′40.3″ N	140°56′40.6″ E	12.5	Non-decontaminated
	(E) Karino	37°30′18.1″ N	140°57′32.9″ E	11.2	Decontaminated
	(F) Tajiri	37°29′10.0″ N	140°57′18.0″ E	9.8	Decontaminated
	(G) Obori	37°28′38.0″ N	140°57′25.3″ E	9.0	Non-decontaminated
	(H) Kakura	37°29′59.4″ N	140°57′47.7″ E	10.5	Decontaminated
	(I) Midorigaoka	37°29′26.7″ N	140°57′41.5″ E	9.8	Decontaminated
	(J) Uenohara	37°29′29.0″ N	140°58′00.8″ E	9.6	Decontaminated
	(K) Sakata	37°30′10.5″ N	140°58′39.1″ E	10.1	Decontaminated
	(L) Kawazoe	37°29′44.1″ N	140°58′53.7″ E	9.3	Decontaminated
	(M) Ushiwata	37°29′11.3″ N	140°58′37.4″ E	8.6	Non-decontaminated
	(N) Nishidai	37°30′08.1″ N	140°59′31.3″ E	9.5	Decontaminated
	(O) Gongendo	37°29′48.2″ N	140°59′32.4″ E	8.9	Decontaminated
	(P) Hiwatashi	37°29′20.5″ N	140°59′34.0″ E	8.1	Decontaminated
	(Q) Takase	37°28′57.3″ N	141°00′01.8″ E	7.2	Decontaminated
	(R) Kiyohashi	37°29′54.9″ N	141°00′59.5″ E	8.5	Decontaminated
Tama area, Tokyo	Chofu Airport *	35°39′58.0″ N	139°31′54.0″ E	236.8	–

* The nearest monitoring post from the sampling point (data was retrieved from https://radioactivity.nsr.go.jp/map/ja/download.html on 15 March 2017). ^†^ This column represents the decontamination status of each sampling point as of 10–11 September 2016.

## Data Availability

The data presented in this study are available on request from the corresponding author.
